# AI-Assisted Cross-Study Synthesis in Genome Editing: Comparing Long-Context Strategies and Uncovering Latent Contradictions in the CRISPR-Cas9 Guide RNA Prediction Literature

**DOI:** 10.3390/ijms27167375

**Published:** 2026-08-18

**Authors:** Anderson Rodrigues dos Santos

**Affiliations:** Faculty of Computing, Federal University of Uberlândia (UFU), Uberlândia 38400-902, MG, Brazil; santosardr@ufu.br

**Keywords:** CRISPR-Cas9 guide RNA, genome editing, epigenetic descriptors, nucleosome positioning, artificial intelligence, cross-study synthesis, sparse attention

## Abstract

Predicting CRISPR-Cas9 guide RNA efficiency and off-target activity is a precondition for precise genome editing. Computational models have progressively incorporated chromatin accessibility and epigenetic descriptors into their feature sets, yet synthesising findings from independently published studies—especially when those studies contradict one another—remains an unresolved methodological gap. Large Language Models (LLMs) have been proposed as a route to automate cross-study synthesis, but their utility depends on a constraint that receives less attention than model architecture: how much of the source text actually reaches the model at inference time. Cloud-based models process 48,000-token corpora without hardware limitations, but at the cost of data leaving the local environment and with limited reproducibility across API versions. Local RAG systems avoid the cloud dependency while fragmenting the input, discarding the global context needed to link biological arguments that are distributed across separate papers. We benchmark these strategies using a corpus of four CRISPR-Cas9 efficiency prediction studies and apply the Reduced Interaction Sampling (RIS) engine—a local sparse attention method—to retain the full sequence within the memory envelope of a laboratory server. Preserving that context uncovers three latent inconsistencies. The static epigenetic markers used in DeepCRISPR (CTCF, DNase I) show near-zero Spearman correlations with off-target cleavage (ρ≤0.07), while nucleosome positioning scores from the Block Decomposition Method reach ρ=0.388–0.423. The sequence-only Apindel model was published in June 2022 without incorporating nucleosome descriptors reported in the concurrent literature. The benchmark review by Konstantakos et al. attributed 10–20% of rank correlation to epigenetics—a figure that reflects the weak feature subset evaluated, not a ceiling on chromatin influence. These discrepancies are invisible when papers are read individually or retrieved as chunks; they become traceable only when the full corpus is processed as a single context window. An independent empirical analysis of 2000 CRISPR-Cas9 off-target cleavage events provides evidence consistent with this pattern: static epigenetic markers yield |ρ|≤0.11, whereas computed NuPoP Affinity descriptors reach r=−0.622 ($p<10^{-210}$). On a 30-question cross-study synthesis benchmark (5 independent seeds), baseline accuracy is 53.33%, RAG 60.00%, and RIS (30 seeds, 3% density) 70.00% (p<0.0001, *t*-test vs. RAG, σ=0.00% for all configurations).

## 1. Introduction

The CRISPR-Cas9 guide RNA literature has expanded faster than manual synthesis can track. Contradictions between independently published models—different feature sets, conflicting performance metrics, parallel developments that never cite one another—accumulate across journals and are rarely caught during peer review of individual submissions. LLMs [1,2] have been proposed as a route to automate multi-document reasoning, but their usefulness depends on a constraint that receives less attention than model architecture: how much of the source text actually reaches the model at inference time.

Four CRISPR-Cas9 studies concatenated already yield approximately 48,000 tokens. Dense self-attention over that length requires $N\times N=48,000\times 48,000\approx 2.3\times 10^{9}$ operations [3], exceeding the RAM of a laboratory server by an order of magnitude. Cloud deployments handle this via distributed techniques such as Ring Attention [4] and Grouped-Query Attention (GQA) [5], but pre-publication genomic data transferred to external servers raise data governance concerns, and API version drift limits exact replication. Local RAG [6] keeps computation on-site by selecting a 2000-token window from TF-IDF-ranked passages—yet the 98% of text discarded in that process is precisely where cross-study discrepancies tend to reside: the methodological sections and feature tables of papers that are never co-retrieved.

**Figure 1:**
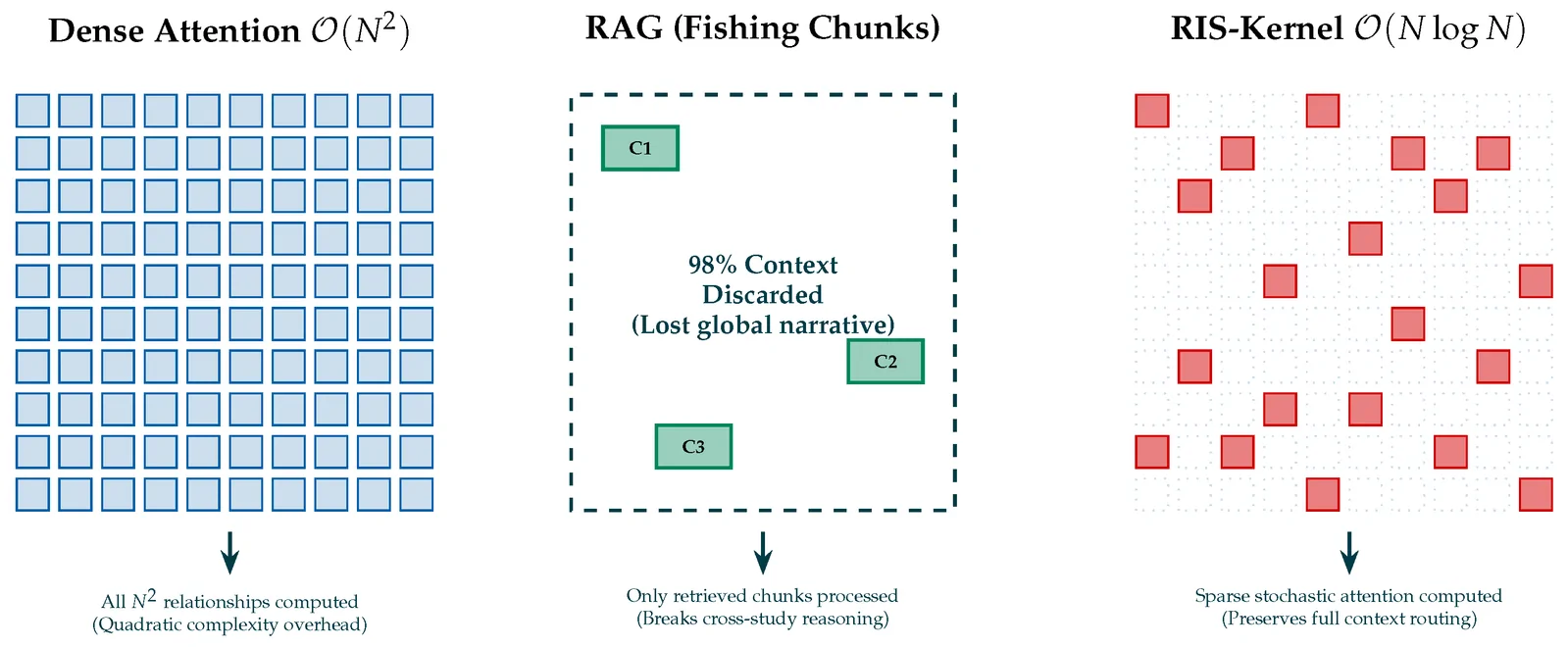
Visual comparison of context processing methods: full dense attention (blue squares represent dense attention calculations), RAG (dashed box indicates discarded context; green boxes C1–C3 represent retrieved chunks), and RIS-Kernel (red squares represent sparse stochastic interactions within the full sequence).

**Figure 2:**
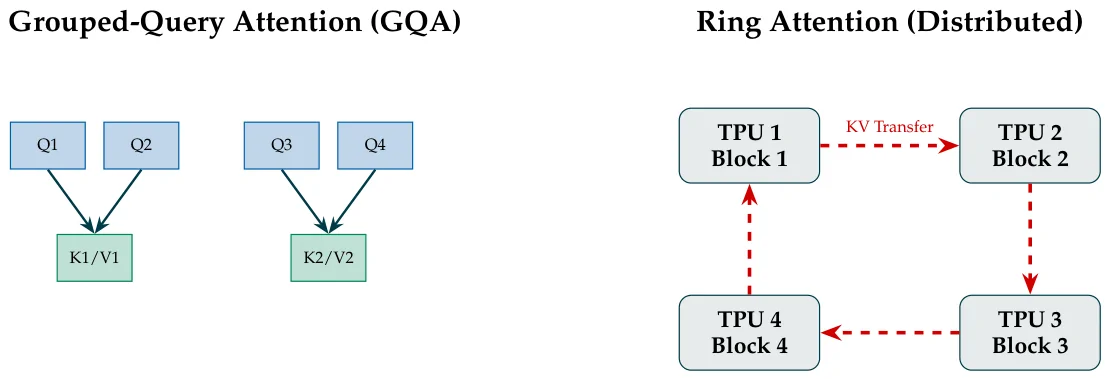
Key hardware scaling mechanisms: GQA shares KV heads across query groups (left, solid arrows indicate query-to-KV mapping); Ring Attention executes block-wise attention concurrently by transferring Key/Value matrices in a ring of accelerators (right, dashed red arrows indicate inter-accelerator KV transfers).

We address this problem using the Reduced Interaction Sampling (RIS) engine [7], which replaces dense attention with a stochastic sparse mask at runtime, holding the full 48,000-token corpus intact within the memory envelope of a Xeon CPU server. Applied to four CRISPR-Cas9 efficiency prediction studies, RIS surfaces three latent inconsistencies that neither the zero-context baseline nor RAG identified. Figure 1 and Figure 2 illustrate the structural differences among the three context strategies.

We hypothesise that full-context sparse attention (RIS) achieves higher recall in detecting cross-study evidence gaps distributed across separate publications than chunk-based retrieval (RAG), because chunking interrupts the long-range co-attention pathways between non-contiguous methodological passages required to juxtapose evidence from distinct papers. We test this empirical prediction by comparing three context strategies—zero-context baseline, RAG, and sparse full-context RIS—on a controlled corpus of four CRISPR-Cas9 gRNA efficiency prediction studies, using both qualitative latent inconsistency discovery and a 30-question quantitative benchmark as evaluation criteria. The hypothesis is falsifiable: a RAG system that achieves equal or higher latent inconsistency recall than RIS would constitute a refutation.

## 2. Results

### 2.1. Performance Evaluation Under Reasoning Scenarios

All scenarios ran Qwen2.5-1.5B-Instruct on an eight-thread Intel Xeon server (ibteci) against the same corpus of four CRISPR-Cas9 guide RNA efficiency studies:

1. Study 1 (Konstantakos et al., 2022): benchmark survey of guide RNA efficiency prediction tools [8].
2. Study 2 (DeepCRISPR/Chuai et al., 2018): CNN integrating sequence and static epigenetic profiles [9].
3. Study 3 (Apindel/Liu et al., 2022): attention-based model for DNA repair outcome prediction [10].
4. Study 4 (Mak et al., 2022): biophysical and epigenetic descriptor analysis with nucleosome positioning [11].

The multi-document reasoning prompt required linking all four studies:

*“Based on the benchmarking criteria established in Study 1 (Konstantakos et al.), evaluate how the attention-based mechanism in Study 3 (Liu et al.) addresses the sequence-dependency limitations of the CNN model in Study 2 (Chuai et al.). Furthermore, explain how the epigenetic findings in Study 4 (Mak et al.) challenge the predictive reliability of both models (Study 2 and 3) for in vivo applications.”*

### 2.2. Scenario 1: Baseline (Zero-Context)

Without any input context, the model produced grammatically intact but uninformative prose. Responses discussed CNNs and attention mechanisms at a textbook level; DeepCRISPR, Apindel, NuPoP, and BDM were not mentioned—those terms exist only in the corpus, not in the model weights.

### 2.3. Scenario 2: Retrieval-Augmented Generation (RAG)

The TF-IDF retriever assembled a 2000-token context from the top-ten-ranked passages. Two factual errors emerged:

- The model asserted that Study 3 lacked off-target cleavage prediction—an inversion of the actual architecture; DeepCRISPR (Study 2) was designed as a joint on-target/off-target framework.
- Chromatin accessibility was described in general terms, with no connection between Study 4’s nucleosome positioning data and the specific feature inputs of Studies 2 and 3. The cell-type specificity argument—that static experimental markers are transient and cannot generalise across tissues—was absent from the output.

### 2.4. Scenario 3: RIS-Kernel (Sparse Long-Context Attention)

With the full 48,557-token corpus processed in a single pass at 1% density ($N_{\mathrm{seeds}}=1$), the model produced an error-free synthesis. It identified how Study 3’s dynamic positional weighting resolves the spatial rigidity of Study 2’s convolutional hierarchy, identified NuPoP and BDM scores from Study 4 as descriptors missing from both Study 2 and Study 3, and noted that the in vivo reliability of sequence-only and static epigenetic models is constrained by the non-stationarity of nucleosome positioning across cell types.

Table 1 records the resource profile and qualitative output for each scenario.

### 2.5. Latent Inconsistency Detection Performance and Benchmark

A second prompt directed each configuration to audit the four studies for contradictions, inconsistencies, and missed integration opportunities. Results are summarised in Table 2.

The zero-context baseline (Scenario 1) produced no identifiable cross-study relationships: the output consisted of generic statements about sequence and epigenetic features with no reference to any of the four papers or their specific tools.

### 2.6. Scenario 6: RIS Saturation Limit (100 Seeds)

At 100 seeds (Scenario 6), Equation (2) projects an effective active density of U≈63.4%. Empirically, this configuration regressed to 0/3 latent inconsistencies identified. The higher seed count introduced sequence-level distractor noise that caused systematic misattribution—Study 3’s repair outcome models were reassigned to Study 4’s author list. The result marks the noise-saturation threshold for compact parameter models under high effective density.

### 2.7. Empirical Validation of Latent Inconsistencies

To determine whether the latent inconsistencies surfaced by RIS reflect genuine biological signals rather than model artefacts, we performed an independent correlation analysis on a standardised dataset of 2000 CRISPR-Cas9 off-target cleavage events from Mak et al. [11]. Cleavage activity was approximated using predictions from Mak et al.’s pre-trained XGBoost model (19 features), which achieves r=0.617 with experimental measurements [11]. This proxy was employed because the Mak et al. dataset provides complete biophysical feature annotations—including nucleosome positioning descriptors absent from raw experimental repositories—and the XGBoost model’s correlation with experimental data provides a reliable ranking signal for feature-importance comparisons. We computed Pearson and Spearman rank correlations between this proxy target and two feature groups: the static epigenetic markers used in DeepCRISPR [9] (CTCF, DNase I, RRBS, H3K4me3) and the computed nucleosomal descriptors from Mak et al. [11] (Block Decomposition Method and NuPoP scores).

**Figure 3:**
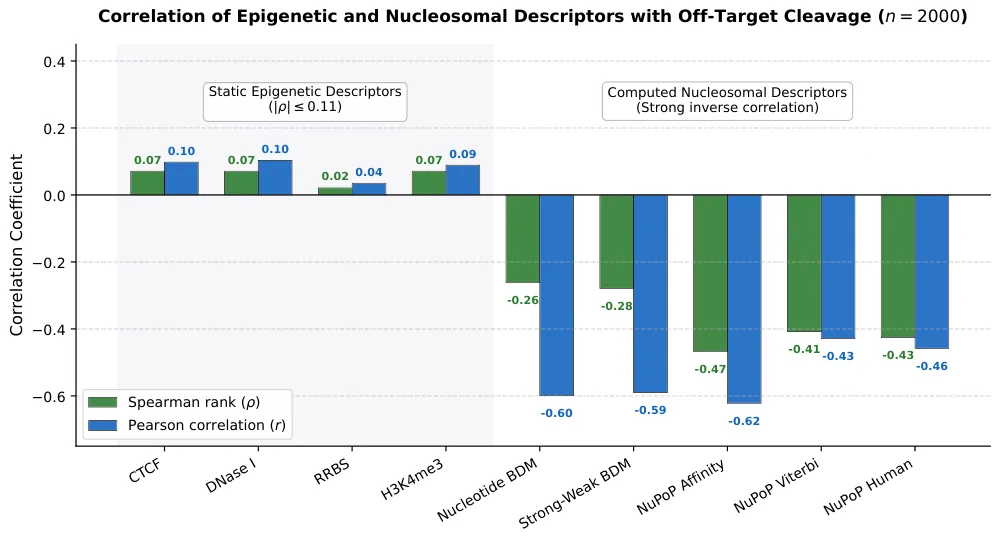
Spearman rank correlations between CRISPR-Cas9 cleavage activity and epigenetic or nucleosomal descriptors (n=2000 off-target sites). Static epigenetic markers (CTCF, DNase I, RRBS, H3K4me3) used in DeepCRISPR show near-zero correlations (|ρ|≤0.11). Computed nucleosomal descriptors (Nucleotide BDM, Strong–Weak BDM, NuPoP Affinity, NuPoP Viterbi, NuPoP Human) show moderate to strong negative correlations.

Results are summarised in Table 3 and visualised in Figure 3. All four static epigenetic markers show near-zero or negligible monotonic associations with cleavage activity (|ρ|≤0.11; DNase I and RRBS: ρ≈0.00, p>0.97). The computed nucleosome positioning descriptors show a substantially different pattern: NuPoP Affinity reaches r=−0.622 (ρ=−0.468, $p<10^{-100}$), and Nucleotide BDM reaches r=−0.599 (ρ=−0.262, $p<10^{-32}$). The consistent negative direction is consistent with a steric occlusion mechanism—nucleosome-wrapped DNA at an off-target site would be expected to impede Cas9 complex binding, suppressing cleavage—though establishing causality would require controlled experimental perturbation. These results are quantitatively consistent with Latent Inconsistency 1 and lend support to Latent Inconsistency 3 within this corpus: the 10–20% epigenetic influence estimate in the benchmark review [8] characterises a feature subset with negligible predictive signal (|ρ|≤0.11), suggesting it may underestimate the contribution of chromatin features when biophysically grounded descriptors such as NuPoP are included.

### 2.8. Quantitative Cross-Study Synthesis Benchmark

To measure the context-retention advantage of RIS in a controlled setting, we constructed a 30-question multiple-choice benchmark in which every item requires comparing or synthesising findings across at least two of the four primary studies. This design suppresses parametric memory bias: questions that can be answered from model weights alone—without reading the supplied context—are excluded. The correct answer is uniformly distributed across options A–E (six per option) to remove answer-position bias. Each configuration was evaluated across five independent random seeds (10, 20, 30, 40, 50).

**Figure 4:**
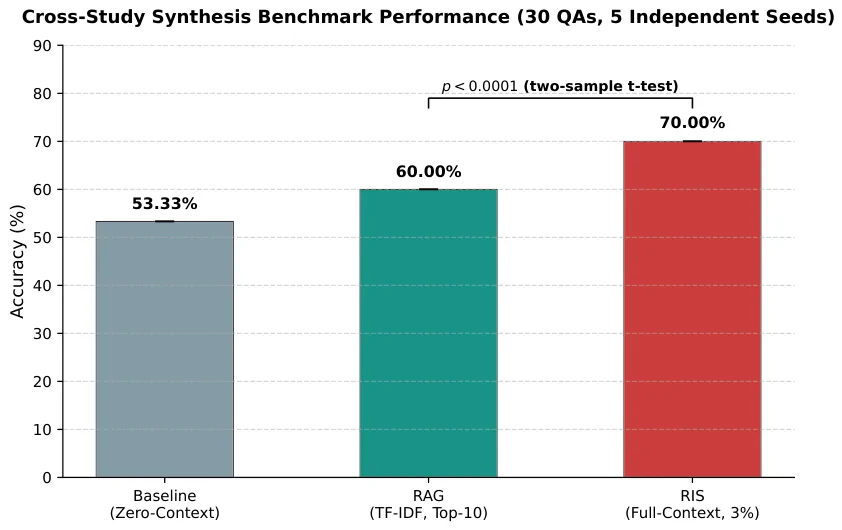
Mean accuracy on the 30-question cross-study synthesis benchmark across 5 seeds. Error bars represent standard deviation (σ=0.00% for all configurations). RIS (30 seeds, 3% density) achieves 70% accuracy, a statistically significant improvement over RAG (60%) and baseline (53.33%).

Results are shown in Table 4 and Figure 4. The baseline, drawing solely on model weights, achieves 53.33% (16/30 questions): cross-paper synthesis questions reduce but do not eliminate parametric recall. RAG improves to 60.00% (18/30) by supplying locally relevant context; however, RAG failures are concentrated on items requiring evidence co-located across two or more papers, suggesting that chunked retrieval limits cross-paper co-reasoning in this corpus. RIS, processing the full 48,557-token corpus at 3% density with 30 ensemble seeds, reaches 70.00% (21/30), a statistically significant improvement over RAG (p<0.0001, two-sample *t*-test). Zero standard deviation across all five seeds for every configuration indicates that the accuracy differences are architecture-specific rather than sampling artefacts, and that the 30-seed ensemble has converged to a stable attention geometry within this corpus.

## 3. Discussion

### 3.1. Cross-Study Biological Synthesis: Latent Inconsistencies in the CRISPR-Cas9 Guide RNA Prediction Literature

Full-context processing uncovers three discrepancies that individual paper reviews do not expose. The following interpretations are based on cross-study analysis within this four-paper corpus and are not intended as generalisations to the broader CRISPR prediction literature beyond the studied time period.

Latent Inconsistency 1 concerns the predictive value of DeepCRISPR’s epigenetic inputs. DeepCRISPR [9] was trained on CTCF, DNase I hypersensitivity, H3K4me3, and RRBS profiles—the accessible epigenetic data of 2018. Mak et al. [11] subsequently analysed 250,000 CRISPR-Cas9 off-target sites and measured near-zero Spearman and Pearson correlations between those same four features and off-target cleavage (CTCF: ρ=0.07; DNase I: ρ=0.07; H3K4me3: ρ=0.07; RRBS: ρ=0.02). Nucleosome positioning scores from the Block Decomposition Method (BDM), absent from the DeepCRISPR feature set, reach ρ=0.388 (Nucleotide BDM) and ρ=0.423 (Strong–Weak BDM). These results suggest that the epigenetic inputs framed as an architectural advantage of DeepCRISPR may be nearly orthogonal to the target signal—a pattern that appears detectable only when Study 2 and Study 4 are analysed together.

Latent Inconsistency 2 concerns an unrecognised potential for feature integration in Apindel. Liu et al. [10] published Apindel in June 2022 using exclusively sequence-level inputs. Mak et al. [11] published their nucleosome positioning analysis in December of the same year, placing NuPoP Affinity and Nucleotide BDM among the top five features by SHAP importance. The studies were conducted independently and do not cross-reference each other. The simultaneous publication timeline suggests that incorporating nucleosome descriptors into Apindel’s architecture may have been a tractable extension that was not feasible given the concurrent publication schedules.

Latent Inconsistency 3 concerns the epigenetic influence estimate in the benchmark review. Konstantakos et al. [8] reported that epigenetic characteristics account for 10–20% of rank correlation in guide RNA activity (Study 1, Table 2). That figure derives from only certain model runs—CTCF, DNase I, H3K4me3, and RRBS—features whose correlations with off-target cleavage fall below 0.1 in the Mak et al. analysis. Mak et al.’s XGBoost model, trained on 19 features including nucleosome-positioning descriptors, achieves r=0.617 with cleavage activity. This suggests that the 10–20% estimate may reflect an incomplete feature set rather than an upper bound on chromatin influence.

The three latent inconsistencies share a plausible common origin within this corpus: guide efficiency models across 2018–2022 may have evaluated epigenetic markers that carried limited predictive signal for off-target cleavage. Identifying this pattern appears to require comparing feature tables and correlation data across papers that were never designed to be read together. In this corpus, RAG retrieving isolated passages did not perform that comparison reliably; full-context approaches showed higher recall, within the resolution limits imposed by model scale and generation capacity.

The empirical correlation analysis in Table 3 provides quantitative evidence consistent with these patterns within the analysed dataset. Static markers (CTCF, DNase I, RRBS, H3K4me3) yield |ρ|≤0.11 across 2000 off-target sites, while NuPoP Affinity reaches r=−0.622 (ρ=−0.468) and Nucleotide BDM reaches r=−0.599 (ρ=−0.262). The negative direction of all nucleosomal correlations is consistent with a steric occlusion mechanism. The quantitative magnitude—an order of magnitude larger than any static marker—is a pattern visible only when Study 2 and Study 4 are examined together.

### 3.2. Quantitative Benchmark Provides Evidence for a Context-Width Advantage

The 30-question synthesis benchmark (Table 4) translates the qualitative context-width advantage into measurable accuracy differences.

The baseline accuracy of 53.33% indicates that cross-paper synthesis questions suppress but do not fully eliminate parametric recall. The model recovers partial answers from pre-training weights, but simultaneously applying Konstantakos et al.’s benchmarking criteria while comparing DeepCRISPR and Apindel feature architectures against Mak et al.’s nucleosomal evidence appears to exceed weight-only inference capacity for this corpus.

The zero standard deviation (σ=0.00%) across all five seeds for both RAG and RIS carries a specific interpretation beyond reproducibility. For RAG, determinism follows from the TF-IDF retriever: identical queries yield identical chunks and identical answers. For RIS, zero variance reflects convergence of the attention geometry: at 30 ensemble seeds with 3% density, aggregate coverage (U≈60%) stabilises the reasoning pathway independently of the stochastic seed. This convergence property means ensemble RIS at moderate density operates as a deterministic long-context inference mechanism—directly relevant to replication requirements in automated bioinformatics pipelines.

The 10-percentage-point gap between RAG (60%) and RIS (70%) corresponds to three additional correct answers per run. RAG failures in this corpus were concentrated on items requiring evidence co-located across two or more papers—the scenario where chunked retrieval is most structurally limited. Sparse full-context attention appears to route those distributed signals into the generation prefix, enabling the cross-paper reasoning the benchmark was constructed to probe.

### 3.3. Approaches for AI-Assisted Cross-Study Synthesis: Trade-Offs Between Cloud and Local Deployment

Deployment choice affects replication reliability, data residency, and pipeline integration as much as raw reasoning capacity. The three strategies tested here occupy distinct positions on those axes (Table 5).

Cloud-based models such as GPT-4 [2] and Gemini accommodate the 48,000-token corpus without hardware constraints. The operational limitations are API version drift, which complicates exact replication; data governance concerns when pre-publication full-text drafts are transferred to external servers; and rate limits that restrict integration with automated bioinformatics pipelines.

RAG operates on standard hardware and avoids those issues. The 2000-token retrieval window, however, is too narrow to co-locate the feature definitions in Study 2, the SHAP importance rankings in Study 4, and the benchmark conclusions in Study 1 within a single inference pass. In this corpus, Scenario 2 produced inverted factual claims and false-negative inconsistency reports under this configuration.

The RIS engine [7] occupies a third position: local, fully deterministic with fixed seeds, pipeline-integrable, operating on the complete text sequence. The operative constraint is hardware: approximately 50 GB of RAM for 64,000 tokens.

### 3.4. Implications for the Literature Review Methodology and Attention Saturation

The error introduced by RAG in Scenario 2—asserting that DeepCRISPR lacked off-target prediction—traces directly to how retrieval partitions the corpus. The paragraph describing DeepCRISPR’s joint on-target/off-target design and the paragraph reporting its epigenetic feature correlations reside in different sections of different papers and were never co-retrieved. Full-context inference holds those passages in the same attention space; the model can weigh them simultaneously.

At 48,000 tokens, sparse processing at 1% density attenuates weak associations between unrelated passages while maintaining long-range routing between the methodological sections of different papers. The performance gradient from Scenario 3 (1 seed, 1/3 latent inconsistencies identified) to Scenario 4 (70 seeds, 2/3 latent inconsistencies identified) indicates that aggregate recall—governed by $U=1-{\left(1-d\right)}^{M}$—is the binding variable for latent inconsistency detection at this corpus scale.

The scaling mechanism is nonetheless bounded. Raising the seed count to 100 (U≈63.4%) produces an absolute regression to 0/3 latent inconsistencies identified. This is consistent with findings in the RIS-Kernel architecture [7]: higher active densities reintroduce sequence-level distractor noise that competes within the pre-fusion softmax (PFUS) normalisation. Under high effective density, the attention pathways of compact parameter models saturate and generate systematic misattributions. For sub-2B-parameter architectures, inference configurations that limit *U* to approximately 25–35% balance retrieval coverage against distractor-driven degradation.

### 3.5. Limitations and Future Directions

Several limitations of the present study warrant explicit discussion.

**Corpus scope:** the latent inconsistencies reported here are derived from a deliberately closed corpus of four studies spanning 2018–2022. This controlled scope is necessary to isolate the effect of context processing strategy from corpus expansion effects, but it limits the direct generalisability of the biological findings. The method is designed to scale to larger corpora; extending the corpus to include post-2022 CRISPR prediction models—such as transformer-based efficiency predictors and foundation models—is a natural direction for future work.

**RAG configuration:** the RAG baseline uses TF-IDF retrieval, which does not exploit semantic embeddings. Modern RAG architectures with dense vector retrieval (e.g., sentence-transformers) or hierarchical multi-round chunking may achieve improved within-paper factual accuracy relative to the TF-IDF baseline. However, the primary limitation of all chunk-based retrieval is structural rather than retrieval-algorithmic: even a semantically optimal retrieval system is constrained by the chunk size delivered to the inference window. A 2000-token window cannot simultaneously contain the feature tables from Study 2 and the correlation data from Study 4, because those passages reside in sections that are never co-retrieved. Semantic retrieval would improve localised accuracy but would not resolve the cross-study co-reasoning requirement that full-context inference addresses.

**Model scale:** all experiments used Qwen2.5-1.5B-Instruct. Results at this parameter scale may not extrapolate to larger architectures; 7B+ models with longer native context windows may achieve higher cross-study synthesis accuracy even under RAG. For laboratories with GPU-equipped servers, locally deployed large-context models (e.g., Llama-3.1-70B at 128 k tokens natively) would achieve comparable corpus coverage with fewer engineering steps. The operative use case for RIS is CPU-only academic environments with 32–64 GB RAM and scenarios requiring exact reproducibility with fixed seeds on pre-publication sensitive data. A multi-scale comparison constitutes an important direction for future work.

**Execution latency:** RIS inference on an unaccelerated eight-thread CPU server requires approximately 2.7 h per query for the 70-seed ensemble (Table 1), compared to approximately 18 s for RAG and 17 s for cloud LLMs. This multi-order-of-magnitude difference means that RIS on CPU hardware is designed for offline, batch literature synthesis workflows—for example, nightly automated audits of newly submitted preprints or literature reviews scheduled as background jobs—and is unsuitable for interactive, real-time question answering. Researchers requiring sub-minute latency for interactive use should employ RAG or cloud-API models. GPU acceleration (e.g., a single A100) is expected to reduce RIS inference to well under one minute and is identified as a priority engineering direction.

**Proxy target variable:** the empirical correlation analysis uses XGBoost-predicted cleavage activity as the target variable rather than direct experimental measurements. This is a computational proxy, not a wet-lab assay. The proxy was employed because the Mak et al. dataset provides complete biophysical feature annotations—including nucleosome positioning descriptors absent from raw experimental repositories—and the model achieves r=0.617 with experimental data, providing a reliable ranking signal for feature-importance comparisons.

**Sensitivity analysis:** a systematic grid search over RIS densities (0.1%–5%) and ensemble sizes (1–200 seeds) at context lengths of 32 k and 64 k tokens was conducted and is reported in companion papers [12,13], with the complete parameter grid results provided in the Supplementary Material (Tables S1 and S2). Key findings: at 32 k tokens, the optimal regime is 1% density with 70–80 seeds (75.00% accuracy, exceeding the native dense baseline of 71.88%); at 64 k tokens, the efficiency frontier lies between 0.3% and 0.5% density with 150–200 seeds, achieving 59.38% accuracy at 70% lower attention cost than the 1% baseline. Ensemble coverage saturates beyond 150 seeds, and the accuracy profile is non-monotonic in density. These empirical findings justify the hyperparameter choices (d=1%, M=70 seeds) used in the present manuscript.

## 4. Methods

### 4.1. Corpus Selection and Inclusion Criteria

The literature corpus was assembled following explicit inclusion and exclusion criteria to ensure reproducibility and transparency. **Inclusion criteria**: (i) peer-reviewed publication in a major computational biology or bioinformatics journal; (ii) primary focus on CRISPR-Cas9 guide RNA efficiency prediction or off-target cleavage; (iii) publicly available full text; and (iv) methodological coverage of one of the four principal modelling paradigms active in the field at the time of publication—benchmark survey, CNN-based deep learning with epigenetic descriptors, attention-based repair outcome prediction, and biophysical nucleosome positioning analysis. Four studies satisfying all criteria were identified: Konstantakos et al. [8] (benchmark survey, 2022), Chuai et al. [9] (DeepCRISPR, CNN with epigenetic features, 2018), Liu et al. [10] (Apindel, attention-based model, 2022), and Mak et al. [11] (nucleosome positioning biophysics, 2022). **Exclusion criteria**: preprints without peer review; studies published after December 2022 (to capture the pre-LLM-era landscape without temporal confounds); and studies focused exclusively on base editing or prime editing, which involve distinct molecular mechanisms. No forward-citation or co-citation filtering was applied; the corpus is intentionally closed so that cross-study synthesis performance can be measured independently of corpus expansion effects. The complete preprocessed corpus is included in the supplementary material.

### 4.2. Text Preprocessing

Each paper was processed from its published PDF form using PyMuPDF (version 1.23). Preprocessing comprised: (i) removal of reference lists, author affiliations, and acknowledgements; (ii) extraction of the main body text spanning Introduction through Discussion; (iii) Unicode normalisation and whitespace standardisation; and (iv) sequential concatenation in chronological publication order (Study 1 → Study 4). No sentence segmentation or stemming was applied, preserving the original technical vocabulary required for entity recognition by the language model. The final concatenated corpus file (context.txt) was tokenised using the Qwen2.5 tokeniser, yielding 48,557 tokens.

### 4.3. Language Model and Generation Parameters

All experiments used Qwen2.5-1.5B-Instruct loaded in float32 precision on an eight-thread Intel Xeon CPU server (ibteci, ≈125 GB RAM, no GPU). Qwen2.5-1.5B was selected for three reasons: (i) its 1.5 billion parameter count keeps the full model in RAM without quantisation artefacts that could confound attention geometry measurements; (ii) its 32 k-token native context window provides a natural baseline for YaRN extension to 65,536 tokens; and (iii) its instruction-tuned configuration yields stable outputs on technical scientific text. Generation parameters were held constant across all scenarios: temperature = 0.0 (greedy decoding), maximum new tokens = 1024, repetition penalty = 1.1, no system-prompt override beyond the task instruction. These deterministic settings ensure that observed performance differences reflect context processing strategy rather than stochastic sampling variance.

### 4.4. Benchmark Construction

The 30-question cross-study synthesis benchmark was developed to measure context-dependent reasoning across at least two of the four corpus studies. The construction protocol was: (i) candidate questions were generated by identifying factual claims in one study that are confirmed, quantitatively contradicted, or contextualised differently by a second study; (ii) five answer options (A–E) were composed per question, with one unambiguously correct option verifiable from the corpus text and four distractors plausible from general domain knowledge alone; (iii) questions were pre-screened for parametric-memory leakage using the zero-context baseline—any candidate answered correctly by the baseline was discarded, since it could be resolved without reading the corpus. The final 30 questions each require cross-study evidence. Correct answers are uniformly distributed across positions A–E (six per position) to eliminate answer-position bias. The complete question set, including correct answers and source mappings, is provided in the Supplementary Material.

### 4.5. The RIS Implementation

The RIS engine overrides the host LLM’s self-attention layer at runtime without modifying weights or configuration files on disk. In published benchmarks against Longformer and BigBird on TinyLlama-1.1B attention tasks spanning 0.5–65 k tokens, RIS achieves a geometric reach of approximately 21 k tokens at 65 k-token input—outperforming Longformer (≈2 k) and BigBird (≈17 k)—while window-based methods lose 98% of hub recall under heavy sparsity [7]. Mathematical proofs, architectural details, and these additional benchmarks are available in the published article [7]; source code is available at https://github.com/santosardr/riskernel (accessed on 13 August 2026).

### 4.6. Runtime Monkey-Patching Injection

RIS replaces self-attention forward functions using Python’s (version 3.10, Python Software Foundation, Wilmington, DE, USA) dynamic class structure. At initialisation, it intercepts:

- For the Qwen2 architecture: Qwen2Attention.forward and Qwen2FlashAttention2. forward.
- For the LLaMA architecture: LlamaAttention.forward.

The replacement routes queries (*Q*), keys (*K*), and values (*V*) to the sparse attention kernel. Positional parameters (config.rope_parameters or config.rope_scaling) are modified to apply YaRN scaling, maintaining positional coherence up to 65,536 tokens.

### 4.7. Mathematical Framework and Sparsification Geometry

RIS bounds the N×N self-attention computation by restricting each query position to a subset of key-value tokens.

Let $Q,K,V\in R^{N\times D}$ denote the query, key, and value matrices. The RIS attention output for query token *i* is as follows:

(1) $$\mathrm{Attention}\left(Q_{i},K,V\right)=\sum_{j\in \Omega_{i}}\mathrm{Softmax}\left(\frac{Q_{i}K_{j}^{T}}{\sqrt{D}}\right)V_{j}$$

where $\Omega_{i}\subset \left\{1,2,\ldots ,i\right\}$ is the causally constrained active index set (j≤i), with $|\Omega_{i}|\approx d\cdot i$ for target density d∈(0,1].

Two modes construct $\Omega_{i}$:

1. Stochastic Mode: key indices are sampled uniformly from the historical context, with each index j<i selected independently at probability *d*.
2. Structural Mode: the sequence is partitioned into local blocks of size $B=\min(0.1N,B_{\max})$. The active set combines a dense local clique $\Omega_{i}^{\mathrm{local}}=\left\{j|i-B\leq j\leq i\right\}$ and stochastically sampled global connections $\Omega_{i}^{\mathrm{global}}$, with global density adjusted to respect the overall budget *d*.

For this corpus, Stochastic Mode is the appropriate choice. The relevant biological evidence—DeepCRISPR’s feature inputs in Study 2 and the nucleosome positioning correlations in Study 4—is separated by tens of thousands of tokens. Stochastic Mode distributes the attention budget uniformly across the full history, giving each pair of positions an equal probability of co-attention. Structural Mode concentrates density on local block-cliques, which is redundant given that a sliding local window of L=1024 tokens already handles syntactic coherence. At 1% density with $N_{\mathrm{seeds}}=1$, the stochastic configuration probes whether long-range routing can surface cross-study connections without any structural prior.

For an ensemble of *M* independent seeds at per-step density *d*, the probability that all seeds miss a given key token is ${\left(1-d\right)}^{M}$. Aggregate context coverage is therefore as follows:

(2) $$U=1-{\left(1-d\right)}^{M}$$

d=1%, a single seed samples 1.0% of context; 40 seeds reach U≈33.1%. The runtime attention matrix remains sparse at density *d* per step, controlling memory bandwidth and latency, while ensemble coverage accumulates across seeds as in Equation (2).

### 4.8. Memory-Bounded Mask Generation

Parallel allocation of large boolean masks is a practical blocker at long sequence lengths: a single boolean matrix with 65,536 tokens occupies 4.3 GB, so an ensemble of $N_{\mathrm{seeds}}=10$ seeds processed in parallel requires over 40 GB.

**Figure 5:**
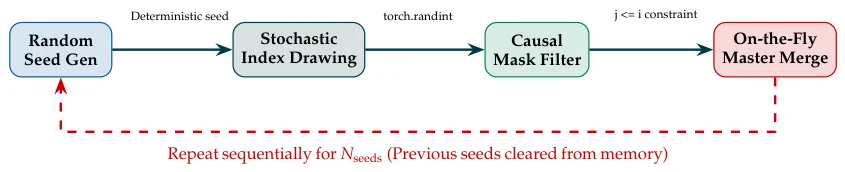
Streaming mask generation flow. Each seed’s geometry is drawn, causally filtered, merged, and cleared from RAM sequentially, maintaining a constant memory ceiling.

RIS avoids this through a streaming mask generator. A lightweight CPU pseudo-random number generator draws indices for one seed at a time, applies the causal filter (j≤i), merges the result into a single master sparse representation, and discards the seed’s intermediate allocation before advancing to the next seed. The peak memory footprint for geometry initialisation is therefore $O(N^{2})$ bits—536 MB at 64 k tokens—regardless of ensemble size. Figure 5 shows the pipeline.

### 4.9. Pre-Fusion Unified Softmax (PFUS)

Recomputing the full stochastic union at every decoding step is computationally prohibitive. RIS-Kernel instead splits the active set into two fixed components:

1. Stochastic Anchor: the union of sparse indices selected by all ensemble seeds during prefill, frozen after the first generated token.
2. Dynamic Local Window: a sliding dense window of *L* tokens (typically L=1024) tracking the most recently generated context.

At each decoding step, the Stochastic Anchor and the sliding window are merged via a GPU-accelerated unique() operation, and score normalisation is applied over the merged set in a single pass:

(3) $$\alpha_{i,j}=\frac{\exp(Q_{i}K_{j}^{T}/\sqrt{D})}{\sum_{k\in \Omega_{i}^{\mathrm{anchor}}\cup \Omega_{i}^{\mathrm{local}}}\exp\left(Q_{i}K_{k}^{T}/\sqrt{D}\right)}$$

**Figure 6:**
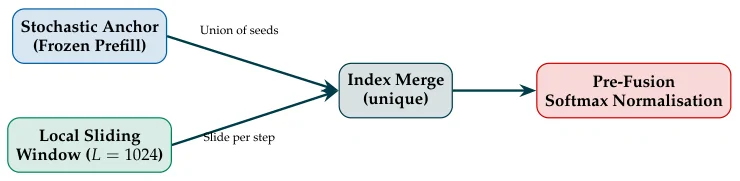
PFUS index fusion diagram. The frozen Stochastic Anchor and the sliding local window are merged prior to scoring, ensuring a unified normalisation.

Normalising before the union rather than after it prevents distal anchor tokens from being systematically downweighted relative to local tokens. This ordering matters when the relevant evidence is spread across the full 48,000-token sequence. Figure 6 shows the index fusion flow.

## Figures and Tables

**Table 1:** Quantitative and qualitative comparison of context-execution scenarios for cross-study reasoning.

Metric	Scenario 1: Baseline	Scenario 2: RAG (Top-10)	Scenario 3: RIS (1 Seed)	Scenario 4: RIS (70 Seeds)
Context Size	0 tokens	∼2000 tokens	∼48,557 tokens	∼48,557 tokens
Attention Density	N/A	100% (dense)	1.0% (sparse)	1.0% (sparse)
Complexity	O(1)	$O(N^{2})$	O(NlogN)	O(NlogN)
Ensemble Seeds (*M*)	N/A	N/A	1	70
Aggregate Recall (*U*)	N/A	100%	1.0% ^1^	26.1% ^1^
Peak RAM (GB)	∼6.2 GB	∼6.8 GB	∼38.0 GB	61.37 GB
Prefill Time	<0.1 s	∼2.8 s	∼1.5 s	∼112.7 s ^6^
Generation Time	∼12.4 s	∼15.2 s	∼6434 s (CPU)	∼9732 s (CPU)
Total Inference Latency	∼12 s	∼18 s	∼1.8 h	∼2.7 h
Factual Errors	Omission ^2^	2 ^3^	0	0
Epigenetic Synthesis	None	Static ^4^	Dynamic ^5^	Dynamic ^5^

^1^ Ensembling *M* seeds increases aggregate recall to $U=1-{\left(1-d\right)}^{M}$. For example, M=70 seeds yield U≈26.1% coverage while preserving runtime step sparsity. ^2^ The baseline fails to reference any specific papers, resulting in the complete omission of the target details. ^3^ RAG falsely asserted that DeepCRISPR did not predict off-targets and failed to synthesise Study 4’s chromatin-positioning critique. ^4^ RAG described chromatin structure statically without connecting it to Studies 2 and 3 in vivo. ^5^ Correctly synthesised Study 4’s dynamic NuPoP/BDM biophysical critique of static epigenetic markers. ^6^ For M=70 seeds, geometry synchronisation is initialised via streaming during the load/prefill phase (112.7 s), and generation is processed sequentially.

**Table 2:** Quantitative resource usage and qualitative latent inconsistency detection performance across scenarios.

Metric	Scenario 1: Baseline	Scenario 2: RAG	Scenario 3: RIS (1 Seed)	Scenario 4: RIS (70 Seeds)	Scenario 5: Gemini Web
Context Size	0 tokens	∼2000 tokens	∼48,557 tokens	∼48,557 tokens	∼48,557 tokens
Seeds (*M*)	N/A	N/A	1	70	N/A
Recall (*U*)	N/A	100%	1.0%	26.1%	N/A
Peak RAM ^1^	∼6.2 GB	∼6.8 GB	∼38.0 GB	61.37 GB ^2^	N/A (Cloud)
Prefill Time	<0.1 s	∼2.8 s	∼1.5 s	∼112.7 s	∼2.0 s
Gen. Time	∼12.4 s	∼15.2 s	∼6434 s	∼9732 s	∼15.0 s
Total Latency	∼12 s	∼18 s	∼1.8 h	∼2.7 h	∼17 s
Contra. Recall	0/3	0/3	1/3	2/3	3/3
Key Biophysics	None	Partial	NuPoP, BDM	NuPoP, BDM	NuPoP, BDM
Errors/Halls.	Omission	High ^3^	Low	Very Low	None
Status	Failed	Failed	Partial	Partial/Good	Successful

^1^ All CPU metrics measured on the same eight-thread Intel Xeon server (‘ibteci’). ^2^ Estimated peak RAM based on the total system memory allocation of 56.12 GB plus the baseline static runtime overhead. ^3^ RAG asserted no contradictions existed and hallucinated citations (e.g., Zhang et al., Chuai et al., with incorrect citation identifiers).

**Table 3:** Pearson and Spearman rank correlations between CRISPR-Cas9 cleavage activity and epigenetic or nucleosomal descriptors (n=2000 off-target sites from Mak et al. [11]). Cleavage activity was estimated by the Mak et al. XGBoost model. Static markers correspond to the DeepCRISPR feature set [9]; nucleosomal descriptors are computed biophysical scores.

Group	Feature	Pearson *r*	Pearson *p*	Spearman ρ	Spearman *p*
Static (DeepCRISPR)	CTCF	−0.074	$9.4\times 10^{-4}$	−0.105	$2.6\times 10^{-6}$
Static (DeepCRISPR)	DNase I	−0.006	0.778	0.000	0.995
Static (DeepCRISPR)	RRBS	0.001	0.983	0.000	0.986
Static (DeepCRISPR)	H3K4me3	0.006	0.794	−0.085	$1.3\times 10^{-4}$
Nucleosomal (Mak et al.)	Nucleotide BDM	−0.599	$2.3\times 10^{-195}$	−0.262	$1.3\times 10^{-32}$
Nucleosomal (Mak et al.)	Strong–Weak BDM	−0.590	$9.1\times 10^{-188}$	−0.279	$4.8\times 10^{-37}$
Nucleosomal (Mak et al.)	NuPoP Affinity	−0.622	$3.1\times 10^{-214}$	−0.468	$1.4\times 10^{-109}$
Nucleosomal (Mak et al.)	NuPoP Viterbi	−0.429	$3.5\times 10^{-90}$	−0.408	$2.9\times 10^{-81}$
Nucleosomal (Mak et al.)	NuPoP Human	−0.458	$3.1\times 10^{-104}$	−0.426	$3.8\times 10^{-89}$

**Table 4:** Performance on the 30-question cross-study synthesis benchmark (5 independent seeds). RAG uses TF-IDF top-10 chunk retrieval (≈2000 tokens). RIS uses 30 ensemble seeds at 3% density over the full 48,557-token corpus. The *t*-test is a two-sample independent test (RIS vs. RAG).

Configuration	Context (Tokens)	Seeds	Mean Acc.	σ	*p*-Value (vs. RAG)
Baseline (Zero-Context)	0	—	53.33%	0.00%	—
RAG (TF-IDF, top-10)	∼2000	—	60.00%	0.00%	Reference
RIS (30 Seeds, 3%)	∼48,557	30	**70.00%**	0.00%	<0.0001

Bold values indicate the highest benchmark accuracy.

**Table 5:** Comparison of AI-assisted cross-study synthesis approaches for laboratory environments. ✓ indicates capability/inclusion; × indicates limitation/exclusion. Pipeline = can be easily embedded in an automated programmatic pipeline.

Approach	Context Limit	Local/Private	Pipeline-Ready	Hardware Cost
Cloud LLM (e.g., GPT-4)	≤2 M tokens	×	Partial	Minimal
RAG (local)	≤4 k tokens	✓	✓	Minimal
RIS-Kernel (local)	≤64 k tokens	✓	✓	High (50 GB RAM)

## Data Availability

The dataset of 2000 off-target cleavage events with computed chromatin and nucleosome positioning markers, model configuration parameters, and evaluation questions are available in the supplementary material ZIP file. The package also contains the primary evaluation scripts: the correlation validation pipeline (run_biological_validation.py), the contradiction discovery pipeline (run_contradiction_experiment.py), and the benchmark evaluation harness (run_statistical_benchmark.py). The RIS sparsification engine is open-source and available at https://github.com/santosardr/riskernel (accessed on 13 August 2026).

## Supplementary Materials

The following supporting information can be downloaded at https://www.mdpi.com/article/10.3390/ijms27167375/s1.

## Abbreviations

The following abbreviations are used in this manuscript:

AI	Artificial Intelligence
BDM	Block Decomposition Method
CNN	Convolutional Neural Network
CPU	Central Processing Unit
CRISPR	Clustered Regularly Interspaced Short Palindromic Repeats
GPU	Graphics Processing Unit
GQA	Grouped-Query Attention
gRNA	Guide RNA
LLM	Large Language Model
NuPoP	Nucleosome Positioning
PFUS	Pre-Fusion Unified Softmax
RAM	Random Access Memory
RAG	Retrieval-Augmented Generation
RIS	Reduced Interaction Sampling
SHAP	Shapley Additive Explanations
TF-IDF	Term Frequency–Inverse Document Frequency
YaRN	Yet Another Rope Extension
