# Supplementary material for: AI-Assisted Cross-Study Synthesis in Genome Editing: Comparing Long-Context Strategies and Uncovering Latent Contradictions in the CRISPR-Cas9 Guide RNA Prediction Literature
Source: Int J Mol Sci. 2026 Aug 18;27(16):7375. doi: 10.3390/ijms27167375 (PMC13513003; doi:10.3390/ijms27167375)
Supplement: Supplementary file 1 [file ijms-27-07375-s001.zip › supplementary_material/results/benchmark_stats_synthesis.pdf]

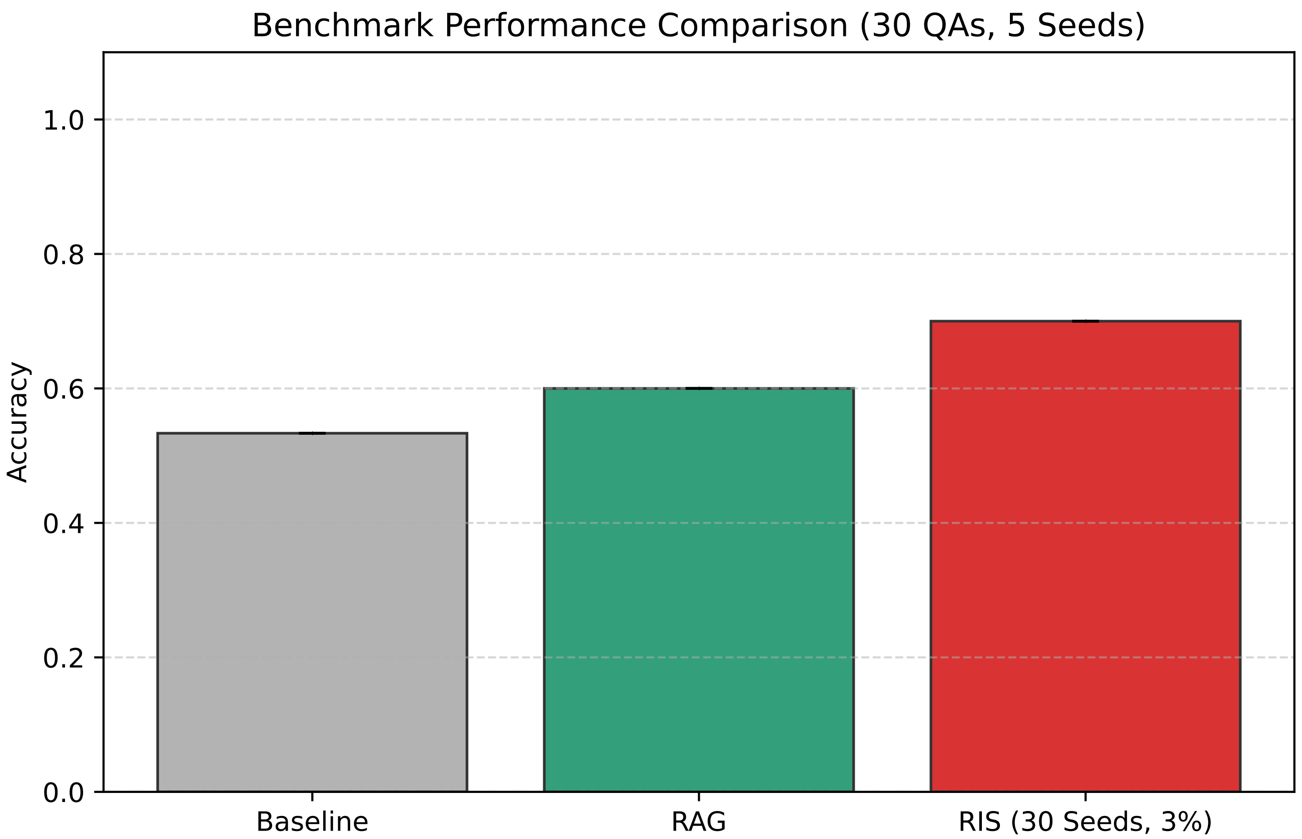
Benchmark Performance Comparison (30 QAs, 5 Seeds)
Accuracy
1.0
0.8
0.6
0.4
0.2
0.0
Baseline
RAG
RIS (30 Seeds, 3%)
