# Supplementary material for: AI-Assisted Cross-Study Synthesis in Genome Editing: Comparing Long-Context Strategies and Uncovering Latent Contradictions in the CRISPR-Cas9 Guide RNA Prediction Literature
Source: Int J Mol Sci. 2026 Aug 18;27(16):7375. doi: 10.3390/ijms27167375 (PMC13513003; doi:10.3390/ijms27167375)
Supplement: Supplementary file 1 [file ijms-27-07375-s001.zip › supplementary_material.pdf]

Supplementary Material: Sensitivity Analysis of Reduced Interaction Sampling (RIS) Hyperparameters

AI-Assisted Cross-Study Synthesis in Genome Editing: Comparing Long-Context Strategies and Uncovering Latent Contradictions in the CRISPR-Cas9 Guide RNA Prediction Literature

Manuscript ID: ijms-4488460

Anderson R. Santos
Faculty of Computing, Federal University of Uberlândia (UFU), Uberlândia, Brazil
santosardr@ufu.br

Overview

This supplementary document provides the full parameter sensitivity grid search data evaluating the Reduced Interaction Sampling (RIS) engine across different attention densities (d), ensemble seed counts (M), and context window sizes (32k and 64k tokens). These empirical data support the hyperparameter selection ($d = 1.0\%$, $M = 70$ seeds) used in the primary manuscript.

Table S1: Sub-1% Density Grid Search (65,536 Tokens)

Table S1 presents the sensitivity grid search for RIS-Structural mode at 65,536 tokens under sub-1% densities ($d \in [0.1\%, 0.9\%]$) using Qwen2-1.5B with YaRN scaling. Baseline zero-context accuracy is 51.56%; native dense accuracy at 1.0% density is 65.63% (100 seeds).

Table S1: Sub-1% density grid search for RIS-Structural at 65,536 tokens. Accuracy for each (density, seeds) combination.

Density (d)	$B_{\max}$	Seeds=50	Seeds=100	Seeds=150	Seeds=200	Best	Mean
0.1%	66	45.31%	45.31%	51.56%	51.56%	51.56%	48.44%
0.2%	131	48.44%	51.56%	54.69%	53.13%	54.69%	51.95%
0.3%	197	48.44%	53.13%	59.38%	59.38%	59.38%	55.08%
0.4%	262	53.13%	54.69%	59.38%	57.81%	59.38%	56.25%
0.5%	328	56.25%	57.81%	59.38%	59.38%	59.38%	58.20%
0.6%	393	54.69%	57.81%	57.81%	56.25%	57.81%	56.64%
0.7%	459	54.69%	56.25%	57.81%	60.94%	60.94%	57.42%
0.8%	524	60.94%	62.50%	57.81%	59.38%	62.50%	60.16%
0.9%	590	54.69%	53.13%	56.25%	57.81%	57.81%	55.47%
1.0%	655	62.50%	65.63%	—	—	65.63%	64.06%

Table S2: Full Parameter Grid Search (32,768 Tokens)

Table S2 details the performance breakdown for both RIS modes (Stochastic and Structural) across densities (1%, 2%, 5%) and seed counts ($M \in [1, 100]$) on the 32,768-token window. Zero-context baseline: 59.38%; Full dense baseline ($w = 32{,}768$): 71.88%.

Table S2: Retrieval accuracy and context gap recovery rate on the 32,768-token window.

Mode	Density (d)	Seeds (M)	Accuracy (%)	Gap Recovery (%)
Zero-Context ($w = 0$)	—	—	59.38	0.0
Full Dense Baseline	100%	1	71.88	100.0
Stochastic	1%	1	59.38	0.0
Stochastic	1%	10	62.50	25.0
Stochastic	1%	20	62.50	25.0
Stochastic	1%	30	62.50	25.0
Stochastic	1%	40	62.50	25.0
Stochastic	1%	50	68.75	75.0
Stochastic	1%	70	75.00	125.0
Stochastic	1%	80	75.00	125.0
Stochastic	1%	90	71.88	100.0
Stochastic	1%	100	71.88	100.0
Stochastic	2%	1	56.25	-25.0
Stochastic	2%	10	65.62	50.0
Stochastic	2%	20	65.62	50.0
Stochastic	2%	30	68.75	75.0
Stochastic	2%	40	71.88	100.0
Stochastic	2%	50	71.88	100.0
Stochastic	2%	60	68.75	75.0
Stochastic	2%	70	71.88	100.0
Stochastic	2%	80	68.75	75.0
Stochastic	5%	1	57.81	-12.5
Stochastic	5%	10	71.88	100.0
Stochastic	5%	80	71.88	100.0
Stochastic	5%	90	71.88	100.0
Stochastic	5%	100	71.88	100.0
Structural	1%	1	59.38	0.0
Structural	1%	10	68.75	75.0
Structural	1%	20	68.75	75.0
Structural	1%	30	68.75	75.0
Structural	1%	40	71.88	100.0
Structural	1%	50	71.88	100.0
Structural	1%	100	71.88	100.0
Structural	2%	1	62.50	25.0
Structural	2%	10	68.75	75.0
Structural	2%	20	68.75	75.0
Structural	2%	30	71.88	100.0
Structural	2%	40	71.88	100.0
Structural	2%	100	71.88	100.0
Structural	5%	1	65.62	50.0
Structural	5%	10	65.62	50.0
Structural	5%	20	68.75	75.0
Structural	5%	80	71.88	100.0
Structural	5%	90	71.88	100.0
Structural	5%	100	71.88	100.0
